# Serum Fibrinogen and Renal Dysfunction as Important Predictors of Left Atrial Thrombosis in Patients with Atrial Fibrillation

**DOI:** 10.3390/jcm12196246

**Published:** 2023-09-28

**Authors:** Karlo Golubić, Petra Angebrandt Belošević, Ana Marija Slišković, Zorana Grubić, Katarina Štingl Janković, Vjekoslav Radeljić, Diana Delić Brkljačić

**Affiliations:** 1 Department of Cardiovascular Diseases, University Hospital Center “Sisters of Mercy”, 10000 Zagreb, Croatia; vjekoslav.radeljic@gmail.com (V.R.); diana.delic@kbcsm.hr (D.D.B.); 2 School of Medicine, Catholic University of Croatia, 10000 Zagreb, Croatia; 3 Department of Cardiovascular Diseases, University Hospital Center Zagreb, 10000 Zagreb, Croatia; petraang37@yahoo.com (P.A.B.); sliskovic_anamarija@yahoo.com (A.M.S.); 4 Department of Biochemistry, University Hospital Center Zagreb, 10000 Zagreb, Croatia; zgrubic@kbc-zagreb.hr (Z.G.); kstingl@kbc-zagreb.hr (K.Š.J.); 5 Department of Biology, Faculty of Science, University of Zagreb, 10000 Zagreb, Croatia; 6 School of Medicine, University of Zagreb, 10000 Zagreb, Croatia

**Keywords:** atrial fibrillation, left atrial thrombus, fibrinogen gene polymorphism, prediction model

## Abstract

Background: As has been shown previously, patients with atrial fibrillation (AF) who have left atrial thrombus (LAT) also have elevated plasma concentrations of fibrinogen. In this study, we tried to determine if this is the consequence of a genetic trait and whether elevated concentrations of fibrinogen could be used to predict LAT in patients with AF. Methods: We recruited 181 consecutive patients scheduled for pulmonary vein isolation (PVI) or direct current cardioversion. The primary endpoint was the presence of LAT on transesophageal echocardiography (TOE). We recorded routine clinical and biochemical data as well as the polymorphism type of the fibrinogen gene for the β chain. To control potentially interfering variables, we performed propensity score matching (PSM). Multivariable and univariable logistic regression models (LRM) were computed using the CHA2DS2-Vasc score, the fibrinogen concentration and creatinine clearance as estimated by the Cockcroft–Gault equation. Results: 60 of 181 patients had LAT as detected by TOE. As expected, patients with LAT had significantly higher concentrations of fibrinogen (3.9 vs. 3.6 g/L); *p* = 0.01 in the unadjusted analysis. After performing PSM, there were no statistically significant differences between the groups, except for creatinine clearance (79.9 vs. 96.8 mL/min); *p* = 0.01. There were also no differences regarding the −455 G/A βfibrinogen polymorphism distribution between the two groups. After constructing the LRM, we found no performance enhancement for the CHA2DS2-Vasc score by adding the fibrinogen concentration or creatinine clearance alone, but when all three variables were put together, there was a significant improvement in LAT prediction (AUC 0.64 vs. 0.72), *p* = 0.026. Conclusion: Our study found no evidence of elevated levels of circulating fibrinogen in patients with LAT or a connection between those levels and the A/A and A positive polymorphism. When used together with renal function markers such as creatinine clearance, plasma fibrinogen concentrations can provide additional power to the CHA2DS2-Vasc score for predicting LAT.

## 1. Introduction

AF is the most common arrythmia in human adults. It is linked to significant mortality and morbidity—primarily due to thromboembolism (mostly stroke)—by promoting left atrial appendage (LAA) thrombus formation [1]. The LAA thrombus is, in turn, thought of as the origin of emboli in thromboembolic complications of AF. This clot formation is often regarded as a surrogate marker for thromboembolic events and presents a contraindication for both DC cardioversions and LA procedures such as pulmonary vein isolation. Currently, the CHA2DS2-VASc score is recommended for thromboembolic risk stratification and for establishing indications for oral anticoagulation in patients with AF [2]. The CHA2DS2-VASc score, however, might not take into account all important factors that raise the likelihood of LAA thrombus development, such as AF type or renal dysfunction. It has also been demonstrated that elevated plasma fibrinogen levels are related to coronary heart disease [2], peripheral artery disease and venous thrombosis [3,4,5]. Based on studies performed until now, 50% of fibrinogen concentration variability is due to genetic factors [3]. Polymorphisms of the β-fibrinogen gene (FGB), including the β −455 G/A polymorphism—which is especially involved in the rate-limiting steps of the formation of the β chain—have been demonstrated to be closely connected to the elevation of plasma fibrinogen levels [4,5]. All of the mentioned factors can contribute to LA thrombus formation, even in the presence of anticoagulation therapy, and it is therefore important to establish the relevance of them all in a single setting. Renal dysfunction also plays an important role, as it influences the normal clot-forming pathways and interferes with anticoagulant therapy.

## 2. Materials and Methods

### 2.1. Patient Population

We included 181 patients who were scheduled for PVI or direct current cardioversion between January 2018 and December 2019 in our university hospital center and who needed a transesophageal echocardiography exam (TOE) prior to the procedure. All patients had a transthoracic echocardiography exam (TTE) performed as well. We divided patients into two groups, one with thrombus in LAA detected on TOE, and the other without thrombus in LAA. Excluding criteria were any prior prosthetic valve surgeries, patients with documented thrombophilia, severe illness, or severe acute infection or active malignant disease. All patients have signed an informed consent form prior to recruitment in the study. The research protocol and the review of EMRs were approved by the ethics committees of the Medical University of Zagreb and the University Hospital Centre Zagreb.

### 2.2. Data Collection

We included demographic data from electronical medical records (EMR; sex, age, and body mass), relevant medical histories (hypertension, diabetes mellitus (DM), stroke/transient ischemic attack (TIA)/thromboembolism, vascular disease (prior myocardial infarction [MI], peripheral artery disease [PAD], or aortic plaque), and congestive heart failure, liver disease, smoking habits (yes/no) and current anticoagulation status). The CHA2DS2-Vasc score was calculated from the relevant variables [2]. TTE data included the left ventricular ejection fraction (LVEF) and left-atrial dilatation as a binary variable. As left-atrial dimensions were not consistently reported in the same manner (usually volume was used, but sometimes also area and diameter), patients were divided into two groups (normal and dilated atria) in accordance with the Guidelines for Chamber Quantification of the European Association of Cardiovascular Imaging [6]. Genetic analysis for −455 G/A β fibrinogen polymorphism was performed to detect 3 genotypes: GG homozygote, GA heterozygote and AA homozygote. The A+ genotype was defined as either GA heterozygote or AA homozygote. The laboratory markers that were used were fibrinogen concentration and serum creatinine. The glomerular filtration rate (GFR) was calculated using the Cockcroft–Gault equation [1,2].

### 2.3. Transesophageal Echocardiography

All patients scheduled for direct current cardioversion for AF or for PVI, who had indications for TOE, were included in the study. Together with TOE, TTE was performed routinely prior to the procedure. TOE examinations are usually conducted within a few hours prior to the scheduled procedure (at most within 24 h before the procedure). All TOE and TTE studies were performed by certified echocardiographers (accreditation in echocardiography by the Section of Echocardiography of the Croatian Cardiac Society), using a VIVID 9 Ultrasound Machine (General Electric, Boston, MA, USA). In cases where the study’s findings were in doubt, they were reviewed by two echocardiographers in order to arrive at a conclusive diagnosis that was the most trustworthy, to enable safe referral for cardioversion or ablation.

### 2.4. Genetic Analysis

All patients had peripheral venous blood samples taken, which were all kept in deep freezers. For DNA extraction, 200 μL of dissolved blood samples were centrifuged at 3000 bpm with a solvent solution for 5 min. For this extraction, we used a commercially available reagent for DNA extraction (Nucleospin Blood, Macherey Nagel, Duren, Germany). The next step was amplification and digestion of the multiplied fragments of the gene promotor-encoding β chain of fibrinogen, which was carried out with the commercially available reagent AMPLI-β FIBRINOGEN −455 G/A (Kit for the detection of −455 G/A polymorphism of the β fibrinogen gene (dia-chem S.R.L, Napoli, Italy).DNA samples were amplified and biotinylated in multiplex polymerase chain reactions (PCR). The amplification conditions were as follows: an initial denaturing step at 95 °C for 5 min, followed by 35 cycles of denaturation at 95 °C for 1 min, annealing at 57 °C for 1 min and extension at 72 °C for 2 min, and a final extension step of 72 °C for 10 min. After this, DNA amplification detection of the −455 G/A polymorphism was performed with the specific restrictive enzyme Hae III. The finished product’s genotypes were examined using a UV transilluminator after electrophoresis on a 1.5% agarose gel.

### 2.5. Study Endpoint

The primary endpoint was the presence of a left atrial thrombus on TOE. As mentioned above, this is frequently employed as a surrogate marker for thromboembolic events, as following hard endpoint outcomes (stroke, TIA or peripheral embolisms) would require substantially larger sample sizes and longer periods of time.

### 2.6. Statistical Analysis

First, we analyzed the unadjusted data in both groups. Categorical and nominal values are presented through corresponding frequencies and shares, and differences between individual groups were analyzed using the χ^2^ test. Continuous values are presented as medians and interquartile ranges, and differences were analyzed using the Mann–Whitney U test. As the study was designed to be observational, we expected significant differences across several variables of interest. In order to counteract those differences, we implemented propensity score matching (PSM) in both groups, using a multivariable logistic regression model with LAT as the outcome, and matched individuals based on their risk score. By compiling a sample of LAT patients that is comparable to a sample of control group patients based on all the observed factors, PSM aims to decrease assignment bias and imitate randomization. After PSM, we were left with two new (matched) groups that could be compared in a case–control type analysis. Then, we analyzed the matched groups using the McNemar test for categorical variables and the Wilcoxon signed ranks test for continuous variables. Lastly, we generated logistic regression models for CHA2DS2-VASc, CHA2DS2-VASc and fibrinogen concentrations;CHA2DS2-VASc and eGFR; and CHA2DS2-VASc, fibrinogen concentration and eGFR. ROC curves were drawn and AUCs calculated and compared using the DeLong method. For all tests, a *p*-value of less than 0.05 was considered significant. All tests were two-tailed. All calculations were performed using MedCalc version 19.2.6 (MedCalc Software, Ostend, Belgium) and Office Excel 2007 (Microsoft, Redmond, WA, USA).

## 3. Results

### 3.1. Patient Characteristics

A total of 181 patients were included, of whom 60 (33.1%) had LAT—predominantly thrombosis of the LAA. The group differences in the unadjusted analysis are described in Table 1.

The demographic factors that were noted did not differ significantly, except in the control group (without thrombus), where there were significantly more men than women (*p* = 0.0027). Patients in the thrombus group had significantly more vascular disease, heart failure and diabetes mellitus (*p* = 0.0042, *p* = 0.0025, and *p* = 0.0484, respectively), compared to the control group, where on the other hand, there were significantly more patients with paroxysmal atrial fibrillation (*p* = 0.0115). The thrombus group had higher fibrinogen levels that were statistically significant compared to the control group, as well as creatinine clearance, which was statistically significantly lower in the thrombus group (*p* = 0.0115 and *p* < 0.001, respectively).

### 3.2. Thromboembolic Risk

Among the LAT patients, 86.7% were regarded as high risk (with a CHA2DS2VASc score ≥ 2) compared to the control group, where only 65.3% patients were of high risk. The LAT and control groups differed significantly from each other in terms of thromboembolic risk; *p* = 0.0025. 

The use of anticoagulant therapy was generally high (over 90% of patients) and did not significantly differ between the two groups. Anticoagulation was mostly achieved by NOACs.

There was no meaningful difference between the two groups in terms of LA size. The global systolic function of the left ventricle (measured as ejection fraction) also did not differ significantly between the two groups. 

There were no differences regarding the prevalence of the homozygous AA genotype between the two groups. When analyzing together with the heterozygous GA genotype (the so-called A+ genotype), we also failed to detect any significant difference between the groups.

### 3.3. Propensity Score Matching (PSM)

A multivariable logistic regression model was computed to perform propensity score matching for the two groups (thrombus and control). Individually obtained risk scores were listed and matched between the two groups. After PSM, we got two new (matched) groups that could be compared in a case–control type analysis that was now adjusted for confounding variables. The adjusted results of the comparison of the 120 patients (60 pairs) are described in Table 2.

No statistically significant changes between the groups were found following PSM, except for creatinine clearance (79.9 vs. 96.8 mL/min; *p* = 0.01). There were also no differences regarding polymorphism distribution between the two groups. 

### 3.4. Logistic Regression Models (LRM) with ROC Curves

New univariable and multivariable logistic regression models were computed with the aim of predicting LAT. In order to assess whether combining CHA2DS2-Vasc with fibrinogen and creatinine clearance would yield a significantly better prediction model, ROC curves were drawn, analyzed and compared with the combined fibrinogen and creatinine clearance models, the CHA2DS2-Vasc model and the model containing CHA2DS2-Vasc, fibrinogen and creatinine clearance. 

The reference univariable model (CHA2DS2-Vasc) had an area under the receiver operating characteristic curve (AUC) of 0.642, with a standard error of 0.0408 and a 95% confidence interval of 0.567 to 0.712.

**Figure 1:**
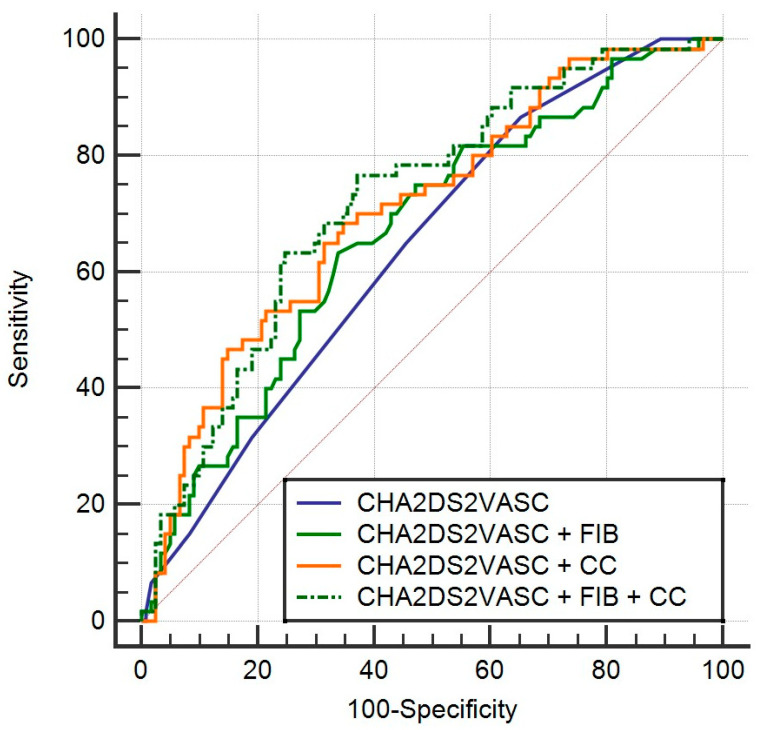
Comparison of ROC curves between the CHA2DS2-Vasc model and CHA2DS2-Vasc +FIB, CHA2DS2-Vasc + CC model and CHA2DS2Vasc + FIB + CC model.

The combined models of only fibrinogen and CHA2DS2-Vascand only creatinine clearance and CHA2DS2-Vasc yielded no significant improvements (AUC differences of 0.0248 and 0.0649; *p* = 0.318 and *p* = 0.054 for pairwise comparison with the reference model, respectively), as seen in Figure 1.

**Figure 2:**
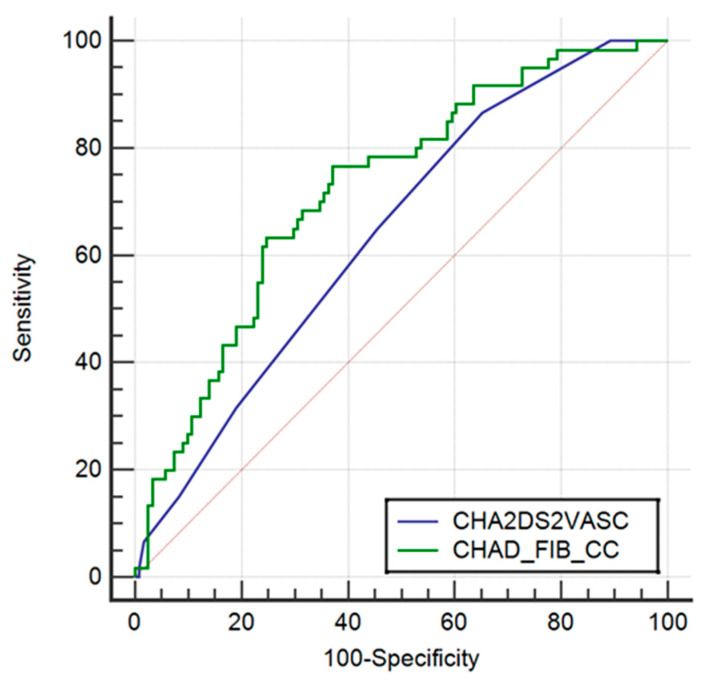
Comparison of ROC curves between the CHA2DS2-Vasc model and CHA2DS2-Vasc +FIB + CC model.

After constructing the LRM containing CHA2DS2-Vasc, fibrinogen and creatinine clearance, there was a significant improvement in prediction (AUC 0.642 vs. 0.723; *p* = 0.026), which is shown in Figure 2.

## 4. Discussion

In our investigation, a very high frequency of LAT was discovered. While there are many possible causes for this—including an extremely sensitive TOE exam, anticoagulant medication failure, biological variability and many others—we think that pure chance is the most plausible explanation, as monotonous series are frequently seen in random walks of a variable. 

Although the current guidelines do not list serum fibrinogen concentration among the factors affecting the probability of LAA thrombus formation, many studies have shown that elevated markers of inflammation and procoagulant states are frequently encountered in LAT [3]. The unanswered question thus far remains whether the aforementioned procoagulant state is the cause or at least a catalyst for LA thrombosis, or could it be that some people (through a possible hyperactivity of the fibrinogen gene) originally have higher serum fibrinogen concentrations that make them more prone to thrombus formation and, consequently, thromboembolic complications from AF. Fibrinogen polymorphism (−455 G/A), as well as other single nucleotide polymorphisms (SNP), are mainly studied in patients with stroke. The exact mechanism by which the −455 G/A fibrinogen polymorphism may affect cardioembolic stroke (CES) pathology remains unknown. Some studies associate high serum fibrinogen concentration and A+ genotype with CES in patients with atrial fibrillation and low thromboembolic risk, as well as with stroke with poor outcomes—mainly in women [4,5].

There are several metanalyses which have associated −455 G/A polymorphism with stroke, which were mostly carried out in the Asian population [6,7,8], and the latest one from 2018 confirmed that −148 C/T and −455 G/A polymorphisms are associated with stroke. Sub analysis revealed that the −455 G/A polymorphism is more often associated with stroke in the Asian population, and on the other hand, −148 C/T polymorphism with stroke in the Caucasian population [9]. These results can partly explain the negative association of FGB polymorphism and LAT in our study, since our population is very homogenous and exclusively of European origin. Based on epidemiological and biochemical studies, one of the most significant genetic polymorphisms linked to an increase in plasma fibrinogen is the 455 G/A polymorphism [10,11]. We found that the patients with LAT had considerably greater amounts of fibrinogen (on unadjusted analysis), which is consistent with findings from earlier investigations. However, after correction for confounding variables, there were no significant differences in serum fibrinogen concentration between the two groups, indicating a probable alternative explanation for the observed phenomenon in terms of a common action of other risk factors. Additionally, we failed to provide evidence to support the association between the −455 G/A polymorphism and thrombotic events, as in some previous studies [12,13,14]. Besides the mentioned polymorphism, there are several other SNPs in the promotor region of β fibrinogen; as such, the functional effect of the−455 G/A polymorphism alone remains controversial. The link between the −455 G/A polymorphism and other causative polymorphisms may be in linkage disequilibrium, which would explain why the −455 G/A polymorphism is associated with increased fibrinogen concentrations. We propose that the discrepancies in the results between studies may result from differences in ethnic background, sample size and other risk factors. 

The next most important predictor of LAT, not included in the thromboembolic risk factors or theCHA2DS2-VASc score, was reduced GFR. Renal impairment has been previously shown to predict thromboembolism in AF [15,16]. In both the ATRIA study and with the R2CHADS2 score, renal dysfunction improved thromboembolic risk stratification [15,17]. Renal dysfunction as well as AF type—two variables not included in thromboembolic risk factors—proved strong, independent predictors of LAT, and might improve thromboembolic risk stratification, according to the new CHA2DS2-VASc-RAF score [18]. It is well recognized that having AF raises the likelihood of having renal impairment, while having AF also accelerates the onset and progression of renal impairment. The risk of thromboembolic cardiovascular adverse events, including all-cause death and stroke, is higher among patients with AF and co-existing renal impairment.

A possible mechanism explaining this association could involve pathophysiological interactions with the natural role of the kidney, as renal dysfunction per se accelerates atherosclerosis, leading to more cardiovascular events and poorer outcomes [19]. AF by itself induces a hypercoagulable state via a number of mechanisms. Disorganized atrioventricular contraction, reduced atrial blood flow, endothelial and endocardial damage and dysfunction, increased tissue factor and von Willebrand factor expression, as well as increased fibrinolysis and platelet activation may be risk factors for the development of thrombi and subsequent systemic emboli. In addition to endothelial damage, altered protein C metabolism, defects in glycoprotein Ib expression, elevated levels of different plasminogen activator inhibitor-1 and von Willebrand factor, abnormalities in various coagulation factor levels and activity and inflammation, renal dysfunction is linked to a prothrombotic state. Additionally, there may be abnormalities in the oxidative, inflammatory or neurohormonal pathways, as well as in the mineral metabolism (such as hyperparathyroidism), which may lead to atherosclerosis and a higher risk of thromboembolic events [19].

Additionally, patients with renal dysfunction are more prone to clotting disorders and bleeding. Furthermore, renal dysfunction also has the potential to interact with anticoagulants and other therapies. This relationship is highly complex, as it is known that some anticoagulants are associated with worsening renal function, while others are dose-sensitive to the estimated glomerular filtration rate. Therefore, renal impairment may heighten the underlying prothrombotic condition through a variety of pathophysiological pathways, increasing the risk of ischemic stroke and other thromboembolisms in AF patients.

The CHA2DS2VASC + FIB + CC score demonstrated good sensitivity and specificity in patients with LAA thrombus (AUC = 0.723; *p*< 0.001), while the CHA2DS2VASC score showed poor sensitivity and specificity (AUC = 0.642), as in previous studies. [20].Even if the suggested score is not yet suitable for use in typical clinical settings, we feel that patients with AF and high fibrinogen levels together with reduced GFR should be treated as a group with higher thromboembolic risk, independently of the calculated CHA2DS2VASC score. The reasons for such a finding are merely speculative at this point. Patients with renal impairment have higher rates of both bleeding and thrombosis, as has previously been demonstrated [21]. Increased bleeding is believed to be primarily caused by platelet dysfunction, with minor contributions coming from anemia’s aftereffects, changes in the coagulation cascade—including aberrant vWF and platelet interactions—elevated nitric oxide and impaired fibrinolysis. Acute coronary syndromes, arteriovenous fistula thrombosis, pulmonary emboli, deep vein thrombosis (DVT) and hemodialysis access catheter thrombosis are examples of increased rates of thrombosis, on the other hand. Although the exact mechanisms causing this pro-thrombotic condition are unknown, hyperfibrinogenemia-mediated enhanced clot strength is a known association [21].

### Strengths and Limitations of the Study

To date, this has been the only study that has looked at the FGB polymorphism’s ability to predict outcomes with regards to LAT with adjustments for not only clinical and echocardiographic risk factors, but also for biochemical markers of inflammation and coagulation at the same time. Only one study has tried to associate FGB with LAA thrombus formation in patients with atrial fibrillation, but the sample size was too small, and the result was negative as well [22]. The next strength of our study is in the fact that the population was rather homogenous regarding anticoagulant therapy; more than 90% of our patients had anticoagulation therapy (91.7% in LAT group, 92.6% in control group) at the time of LAT detection using TOE.

The study we conducted has a number of limitations: Firstly, left atrial thrombus being present on TOE was the main endpoint, (which is a surrogate endpoint) and not ischemic stroke. However, LAA thrombus formation is thought to be the main cause of thromboembolic events in AF patients, making it appropriate to be utilized in risk assessments of AF. Secondly, our study was observational, with a possible selection bias which limits its generalizability, as our database included only hospitalized patients in one hospital center. Additionally, as this was an observational study and we were only able to use the factors that were sampled, additional possible predictors may have gone overlooked. Given that we used PSM, this is especially significant; it balances treatment and control groups on a number of covariates without utilizing a large number of observations and employs a linear combination of factors to obtain a single score (average treatment effect estimate). PSM has the drawback of not accounting for latent traits, but only for observed (and observable) factors. The matching technique cannot take into consideration factors that influence treatment assignment and outcome that cannot be seen. Any concealed bias resulting from latent factors may still be present after matching because the process merely adjusts for observed variables. However, the study’s objective was to identify risk factors that are frequently assessed in clinical practice. There are several studies that have shown seasonal variations in fibrinogen concentration, as well as the fact that fibrinogen concentrations are influenced by many environmental factors (ethnicity, sex, habits such as smoking, physical activity, diet, drugs) [23,24,25,26,27,28]. In our study, we used only one measurement of fibrinogen concentration rather than serial measurements, which would maybe yield different results.

## 5. Conclusions

In the light of already published data, our study provides further evidence of the thrombogenic nature of inflammation markers and renal dysfunction. Two variables not included in the CHA2DS2VASC score (level of serum fibrinogen and renal dysfunction) proved strong predictors of LAT on TOE, and therefore might improve thromboembolic risk stratification in patients with AF. Nevertheless, the proposed CHA2DS2-VASC-FIB-CC score requires validation in further studies.

## Figures and Tables

**Table 1:** Demographic characteristics, comorbidities and clinical thromboembolic risk in unadjusted analysis.

	LAT Group (*n* = 60)				Control Group (*n* = 121)				
			Median	IQR			Median	IQR	*p*
Age			68	60.5–72.5			67	58–72	0.4747
	*n*	%			*n*	%			
Male sex	33	55			93	76.9			0.0027
Smoking	7	11.7			14	11.6			0.9848
AF parox	5	8.3			29	24.0			0.0115
Hypertension	49	81.7			86	71.1			0.1244
DM type II	15	25.0			16	13.2			0.0484
Vascular disease	20	33.3			18	14.9			0.0042
Liver disease	1	1.7			5	4.1			0.3844
CHF	35	58.3			42	34.7			0.0025
CHA2DS2VASc									0.0025
0–1	8	13.3			42	34.7			
2–8	52	86.7			79	65.3			
LA enlargement	52	86.7			96	79.3			0.2293
LVEF			50	40–60			55	45–60	0.0903
FG plasma level			3.9	3.3–5			3.6	3–4.225	0.0115
Anticoagulant	55	91.7			112	92.6			0.8324
CVI/TIA	2	3.3			4	3.3			0.9922
A+	30	50.0			63	52.1			0.7928
A/A	4	6.7			8	13.4			0.092
eGFR Cockroft			79.9	62–97.2			97.3	79.9–112.7	<0.001

Legend: *n*: number of individuals; %: frequency; AF: atrial fibrillation; DM: diabetes mellitus; CHF: chronic heart failure; LA: left atria; LVEF: left ventricular ejection fraction; FG: fibrinogen plasma level; CVI: cerebrovascular ischaemic injury; TIA: transitory ischaemic attack; A+: AA + GA genotype; eGFR: estimated glomerular filtration rate.

**Table 2:** Demographic characteristics, comorbidities and clinical thromboembolic risk in adjusted analysis after PSM.

	Thromb Group (*n* = 60)				Control Group (*n* = 60)				
			Median	IQR			Median	IQR	*p*
Age			68	60.5–72.5			68	62–72	0.9362
	*n*	%			*n*	%			
Male sex	33	55.0			37	61.0			0.125
Smoking	7	11.7			7	11.7			1
AF parox	5	8.3			4	6.7			1
Hypertension	49	81.7			47	78.3			0.5
DM type II	15	25.0			11	18.3			0.125
Vascular disease	20	33.3			16	26.7			0.125
Liver disease	1	1.7			3	5.0			0.5
CHF	35	58.3			38	63.3			0.25
CHA2DS2VASc									1
0–1	8	13.3			9	15.0			
2–8	52	86.7			51	85.0			
LA enlargement	52	86.7			50	83.3			0.5
LVEF			50	40–60			45	37.5–55	0.3524
FG plasma level			3.9	3.3–5			3.6	3.3–4.35	0.4295
Anticoagulant	55	91.7			57	95.0			0.5
CVI/TIA	2	3.3			1	1.7			1
A+	30	50.0			31	51.7			1
A/A	4	6.7			4	6.7			1
eGFR Cockroft			79.9	62–97.2			96.8	79.6–108.7	0.0131

Legend: *n*: number of individuals; %: frequency; AF AF: atrial fibrillation; DM: diabetes mellitus; CHF: chronic heart failure; LA: left atria; LVEF: left ventricular ejection fraction; FG: fibrinogen plasma level; CVI: cerebrovascular ischaemic injury; TIA: transitory ischaemic attack; A+: AA + GA genotype; eGFR: estimated glomerular filtration rate.

## Data Availability

The data sets used and/or analyzed during current study are available from the corresponding author on reasonable request.
